# Adolescent Girls With Type 1 Diabetes Develop Changes in Bone Prior to Evidence of Clinical Neuropathy

**DOI:** 10.1210/clinem/dgae511

**Published:** 2024-07-26

**Authors:** Ivana Shen, Rachel L Usala, Mahshid Mohseni, Mary L Bouxsein, Deborah M Mitchell, Erica L Scheller

**Affiliations:** Division of Bone and Mineral Diseases, Department of Medicine, Washington University School of Medicine, St. Louis, MO 63110, USA; Division of Bone and Mineral Diseases, Department of Medicine, Washington University School of Medicine, St. Louis, MO 63110, USA; Division of Endocrinology, Metabolism, and Lipid Research, Department of Medicine, Washington University School of Medicine, St. Louis, MO 63110, USA; Division of Bone and Mineral Diseases, Department of Medicine, Washington University School of Medicine, St. Louis, MO 63110, USA; Endocrine Unit, Massachusetts General Hospital and Harvard Medical School, Boston, MA 02115, USA; Endocrine Unit, Massachusetts General Hospital and Harvard Medical School, Boston, MA 02115, USA; Division of Bone and Mineral Diseases, Department of Medicine, Washington University School of Medicine, St. Louis, MO 63110, USA; Department of Cell Biology and Physiology, Washington University School of Medicine, St. Louis, MO 63110, USA; Department of Developmental Biology, Washington University School of Medicine, St. Louis, MO 63110, USA; Center of Regenerative Medicine, Washington University, St. Louis, MO 63110, USA

**Keywords:** bone / mineral metabolism, metabolic bone disease, pediatric endocrinology, diabetes, complications, microvascular, neuropathy, type 1 diabetes

## Abstract

**Context:**

Neuropathy and fracture are prevalent complications of type 1 diabetes (T1D). Although correlated in the clinical literature, it remains unknown whether neuropathy contributes to the initiation of bone loss at the earliest stages of disease.

**Methods:**

We performed a single-center, cross-sectional study to quantify parameters of nerve and bone health in adolescent girls with T1D (n = 21) and associated controls (n = 12). Groups were well matched for age, height, strength, and physical activity.

**Results:**

By high-resolution peripheral quantitative computed tomograpy, participants with T1D had lower trabecular bone volume fraction at the distal radius (−14.6%, *P*-adj = .095) and the tibia (−12.8%, *P*-adj = .017) and decreased trabecular thickness (−8.3% radius, *P*-adj = .007; −7.5% tibia, *P*-adj = .034) after adjustment for body size. In the tibia only, cortical bone mineral density was increased by 8.6% (*P*-adj = .024) and porosity was decreased by 52.9% with T1D (*P*-adj = .012). There were no significant differences in bone density by dual-energy x-ray absorptiometry. Participants with T1D also had lower circulating levels of osteocalcin (−30%, *P* = .057), and type I collagen cross-linked C-telopeptide (−36%, *P* = .035), suggesting low bone formation and turnover in T1D. Based on the Michigan Neuropathy Screening Instrument, 9.5% of those with T1D had clinical evidence of diabetic peripheral neuropathy. However, consideration of neuropathy status failed to explain the widespread T1D-associated changes in bone.

**Conclusion:**

Our study defines early deficits in trabecular bone microarchitecture, decreased cortical porosity in the tibia, and suppression of biomarkers of bone turnover in adolescent girls with T1D, prior to the onset of symptomatic peripheral neuropathy. These findings inform our understanding of the rapid progression of skeletal disease in young girls with T1D and suggests that early detection and management strategies may help to prevent fracture and related comorbidities later in life.

Persons with type 1 diabetes (T1D) are recommended for monitoring and management of glycemic control with regular screening as early as adolescence for associated microvascular complications including nephropathy, retinopathy, and neuropathy (1). Bone strength is increasingly recognized as yet another dimension of health potentially compromised in youth with T1D (2-4). In a large population-based cohort study of participants aged 0 to 89 years monitored for 1.9 million person-years, T1D was associated with an increased risk of incident fracture that began in childhood and extended across the lifespan (4). To address this, the American Diabetes Association (ADA) recommends at least 60 minutes of physical activity per day with muscle and bone strength training 3 days/week to support the maintenance and accrual of bone mass among pediatric populations with T1D (1). However, beyond exercise, there are no standards for monitoring or managing diabetic skeletal disease. The paucity of guidelines addressing bone health in pediatric populations with diabetes likely stems from a lack of foundational knowledge, public awareness, and clinical tools to assess the onset, severity, and consequences of early skeletal deterioration.

Pediatric osteoporosis is currently defined by the International Society for Clinical Densitometry based on 2 criteria (5-7). The first is low bone mineral density (BMD) as measured by dual-energy x-ray absorptiometry (DXA); DXA scores are considered significant if less than 2 SDs below children of comparable age, sex, and body size (−2.0 Z-score). The second is the presence of a clinically significant fracture history. This is defined as at least 1 long bone fracture in the lower extremity, at least 2 long bone fractures in the upper extremity, or a vertebral compression fracture with an emphasis on fractures due to low trauma. Diabetic bone disease is not yet clinically distinguished from pediatric osteoporosis, though this may be beneficial in the future to inform treatment. Early identification and management of diabetic skeletal disease are also likely to provide benefits throughout life. Indeed, the potential for permanent disability and even death precipitated by osteoporotic fragility fractures motivates health professionals to develop screening instruments that identify patients with bone changes and high fall risk to create opportunities for interventions that could prevent disease progression and consequent fractures (8-11).

To support bone health in patients with T1D, 1 potential solution is to elucidate the natural course of diabetic complications in and between organ systems. This could facilitate the early detection of developing bone disease and fracture risk based on the standardized annual screening for other complications. One candidate for this is diabetic peripheral neuropathy (DPN). Interest in the relationship between bone and nerve health in T1D is motivated by 2 observations. First, clinical studies show a correlation between DPN, bone loss, and fracture risk in adult populations (12). Second, DPN is becoming increasingly recognized as an early complication of diabetes. Large pediatric cohort studies based on the Michigan Neuropathy Screening Instrument (MNSI) reveal that DPN is already present in 7% of adolescents with T1D and 22% to 39% of adolescents with type 2 diabetes (T2D) (13, 14). Adolescents with T1D also have increases in fracture risk (4). However, it is unclear if bone fragility and DPN co-occur or if the onset of one directly contributes to the onset of the other (eg, neuropathy causing bone loss).

In this report, we conducted a case-control study to investigate the association between changes in bone microarchitecture using high-resolution peripheral quantitative computed tomograpy (HR-pQCT) and BMD by DXA with clinical development of DPN by MNSI in adolescent girls with T1D. Parameters of dyslipidemia, hypertension, muscle strength, and general health were also evaluated. The goal of this study was to provide foundational information regarding the relationship between DPN and bone health at an early stage of disease in adolescents with T1D.

## Methods

### Study Design

This single-center, cross-sectional study included adolescent girls aged 12 to 18 years with duration of T1D ≥ 5 years (n = 21) and associated age- and sex-matched controls (n = 12). Informed consent and assent were obtained from the guardian and participants, respectively, under IRB #201908120. Participants with T1D were recruited from the Washington University diabetes registry and pediatric endocrinology clinics from 2020 to 2022. Exclusion criteria included medical conditions that impact bone metabolism including anemia, osteogenesis imperfecta, fetal alcohol syndrome, leukemia, congenital heart defects, neurological disorders, untreated hyper- or hypothyroidism, rheumatoid arthritis, hyperparathyroidism, epilepsy, Parkinson's disease, cancer, coronary artery disease, peripheral vascular insufficiency, stroke, or premature ovarian failure. In addition, subjects using medications known to impact bone metabolism (bisphosphonates, glucocorticoids, GH) were excluded. Age-matched control participants were enrolled from the community. Control participants did not have any major medical conditions, including those detailed here or active usage of prescription medications.

### Clinical Data Collection

Due to the use of radiation-producing equipment, participation required a negative urine hCG pregnancy test. Height, weight, and blood pressure were measured. We obtained demographic and medical information including self-identified race, current and previous medical conditions, medications, and fracture history. Tanner stage was determined by self-ratings according to a standardized diagram of breast and pubic hair development. Physical activity score was obtained using the Physical Activity Questionnaire for Adolescents through self-reporting (15, 16). The jump test was used as an established, easy-to-administer measure of explosive muscle strength (17). Briefly, with feet together, the participants jumped as far forward as possible. Each participant was permitted to do 3 jumps, and the longest jump was counted.

### Laboratory Data

Fasting serum and plasma samples were obtained in the morning. Comprehensive metabolic panel, hemoglobin A1c (HbA1c), lipid profile, 25-hydroxyvitamin D, and complete blood count with differential were measured by the Core Laboratory for Clinical Studies, a standardized reference laboratory at Washington University. The e601 Vitamin D Total III assay (Roche) is traceable to internal reference standards, which are traceable to the isotope dilution liquid chromatography-tandem mass spectrometry 25-hydroxyvitamin D reference measurement procedure. The isotope dilution liquid chromatography-tandem mass spectrometry is traceable to the NIST Standard Reference Material 2972. Fasting serum samples were stored at −80 °C for serum bone turnover marker evaluation and analyzed in a single batch. Serum osteocalcin (QuidelOrtho Cat# 8001, RRID: AB_3099729) and type I collagen cross-linked C-telopeptide (CTX-1; Immunodiagnostic Systems Cat# AC-02F1, RRID: AB_2923399) concentration were determined by ELISA as directed by the manufacturer. Serum samples for osteocalcin were diluted at a 1:1 ratio with a kit-provided diluent to accommodate expected high osteocalcin levels for adolescent participants.

### High-resolution Peripheral Quantitative Computed Tomography

High-resolution peripheral quantitative computed tomography (HR-pQCT) with the XtremeCT-II system (Scanco) was used to image the distal tibia and distal radius, as described previously (18). To limit bias, the technician analyzing the scan used predefined semiautomated workflows and was blinded to the participant groups. Briefly, tibia and radius length were obtained by a radiology technician by measuring the distal edge of the medial malleolus to the tibial plateau and the distance from the olecranon to the ulnar styloid process, respectively. A total of 168 slices spanning a 10.2 mm region of interest were scanned at the 8% site at the distal tibia and 7% site at the distal radius (offset to center, 5.1 mm above and below) according to the participants’ limb lengths using a 60.7 µm voxel size. Positioning landmarks included the tibial plafond and the medial edge of the distal radius. Scans were reviewed for motion artifact and reimaged if not ≤ 3 on the motion grading scale, consistent with established quality standards (19). The nondominant limb was scanned unless there were prior fractures in the region of interest. In this case, the nonfractured limb was used (occurred in 3 T1D participants, 2 arm/1 leg). Standard evaluation scripts were used for cortical and trabecular bone segmentation and analysis (Cortical bone: sigma 0.8, support 1, lower 450, upper 3000; trabecular bone: sigma 0.8, support 1, lower 320, upper 3000). Micro-finite element analysis was used to estimate failure load in the setting of simulated axial compression (20).

### Dual-energy X-ray Absorptiometry

Whole-body DXA scans (HOLOGIC Horizon A) were obtained for BMD and body composition. A certified radiology technician analyzed the scans, and subtotal Z-scores were determined based on software-provided values (HOLOGIC).

### Neuropathy Clinical Evaluation

The MNSI was used to define the presence of DPN in participants with T1D, applied as used previously for DPN in large pediatric cohorts (13, 14, 21). To minimize variability, this exam was performed by the same individual for all participants after training and practice in consultation with the Feldman Lab at the University of Michigan. The instrument consists of a 15-question self-administered survey (MNSI survey) and an 8-point foot examination by a trained individual that included measures of foot inspection, vibration sensation, and ankle reflexes (MNSI exam). Diagnosis of DPN based on the MNSI in youth with T1D requires a survey score > 4 and/or an exam score ≥ 2 (13, 14). A separate monofilament exam was also performed using a 10-gram monofilament applied 10 times in staggered intervals to the dorsum of the great toe midway between the nail fold and the joint. The participant, with eyes closed, was instructed to respond “yes” each time they felt the filament touch the toe. Monofilament response was graded as normal (correct response to 8 to 10 applications), reduced (correct response to 1 to 7 applications), or absent (no response/sensation).

### Statistical Analysis

This study was powered to detect changes in bone microarchitecture using population standards established for HR-pQCT in adolescents at the same percent site (18). As estimated for 2 independent study groups with continuous means, a sample size of n = 12 to 22 per group is sufficient to detect a 10% to 20% change in most parameters related to trabecular microarchitecture and both trabecular and cortical BMD by HR-pQCT. Power analyses suggest that this sample size is insufficient to detect minor changes in cortical thickness, cortical porosity, or bone strength—unless these changes exceed the 20% threshold. Study data were managed using REDCap. Analyses were performed using SPSS 28.0 (IBM) and R software Version 2023.03.1 (R Foundation for Statistical Computing, Vienna, Austria). Categorical variables are reported as percentages and compared using Fisher's exact test. Continuous demographics, health history, and lab values are reported as mean ± SD and compared using a 2-tailed *t*-test for normally distributed variables and Mann–Whitney-U test if not normally distributed. Multivariable regression was used to adjust bone parameters for body mass index Z-score (BMIz) as a composite index of height and weight that is scaled for age. A Pearson correlation coefficient was calculated to understand the linear correlations between bone turnover markers. A *P*-value of <.05 was considered statistically significant with a *P* < .10 interpreted as a trending result.

## Results

### Characteristics of the Control and T1D Study Population

Table 1 shows the demographics and baseline health information of the 33 participants included in the analysis (n = 12 control, 21 T1D). Groups were well matched for age (control 14.7 ±2.3 vs T1D 15.1 ±2.0 years, *P* = .551), height (control 162.8 ±5.8 vs T1D 162.0 ±10.9 cm, *P* = .520), physical activity (control 2.3 ±0.7 vs T1D 2.5 ±0.7 activity score, *P* = .477), and explosive muscle strength (control 161.2 ±23.6 vs T1D 149.3 ±35.9 cm, *P* = .313). Age at menarche and Tanner stage were also similar between groups, suggesting comparable pubertal development. Most participants in both groups self-identified as White or Caucasian. There was an increased, but not statistically significant, prevalence of fracture in participants with T1D vs controls: 48% and 33% respectively (*P* = .424). The average duration of diabetes was 8.5 ±3.2 years for subjects with T1D. Participants with T1D had significantly higher Z-scores for weight (control 0.40 ±0.97 vs T1D 1.04 ±0.68, *P* = .031) and body mass index (control 0.17 ±0.98 vs T1D 1.04 ±0.74, *P* = .007). Increased body mass was associated with significant increases in percent body fat as measured by whole-body DXA scan (control 35.4%±5.6 vs T1D 38.6%±5.6, *P* = .008). Increased body mass has been linked to the anabolic and fat storage promoting effects of supplemental insulin (22, 23) and is common among adolescents with T1D (2, 24, 25). Diastolic blood pressure was also significantly higher in T1D participants (control 70 ±6 vs T1D 76 ±6 mmHg, *P* = .003) (Table 1).

**Table 1.** General characteristics of the study population

Characteristic	Control (n = 12)	T1D (n = 21)	*P*
Age (years)	14.7 ± 2.3	15.1 ± 2.0	.551
Height z-score	0.53 ± 0.89	0.25 ± 1.34	.520
Height (cm)	162.8 ± 5.8	162.0 ± 10.9	.736
PAQ-A activity score (1–5)	2.3 ± 0.7	2.5 ± 0.7	.477
Explosive muscle strength (cm)	161.2 ± 23.6	149.3 ± 35.9	.313
Age at menarche (years)	11.8 ± 1.2 (n = 9)	12.2 ± 1.0 (n = 19)	.334
Premenarche, n (%)	3 (25)	2 (9.5)	.242
Birth control usage, n (%)	0 (0)	4 (19)	.146
Tanner Stage 3, n (%)	3 (25)	7 (33)	.463
Tanner Stage 4, n (%)	5 (42)	12 (57)	.481
Race, White,*^a^* n (%)	11 (92)	20 (95)	.602
History of fracture, n (%)	4 (33)	10 (48)	.424
Duration of diabetes (years)	n/a	8.5 ± 3.2	n/a
**Weight z-score**	**0.40 ± 0.97**	**1.04 ± 0.68**	**.031**
Weight (pounds)	123.8 ± 33.7	145.6 ± 37.1	.013
**BMI z-score**	**0.17 ± 0.98**	**1.04 ± 0.74**	**.007**
**BMI**	**21.1 ± 5.2**	**25.1 ± 5.1**	**.003**
Subtotal fat mass (kg)	19.4 ± 9.2	24.9 ± 10.1	.075
**Subtotal body fat (%)**	**35.4 ± 5.6**	**38.6 ± 5.6**	**.008**
Subtotal Lean Mass (kg)	32.7 ± 7.4	36.7 ± 7.4	.148
***Systolic blood pressure (mm Hg)***	***112 ± 9***	***118 ± 11***	***.094***
**Diastolic blood pressure (mm Hg)**	**70 ± 6**	**76 ± 6**	**.003**

Data are expressed as mean ± SD or numbers (*n*). Values of *P* were calculated by 2-tailed *t-*test and Mann–Whitney U-test in case of continuous variables or Fisher's exact test in case of discrete variables. Significant *P* < .05 values are shown in bold, trending *P* < .01 in italicized bold. Data presented as mean ± SD unless otherwise indicated.

Abbreviations: BMI, body mass index; n/a, not available; PAQ-A, Physical Activity Questionnaire for Adolescents; T1D, type 1 diabetes.

^*a*^Only 1 control and 1 participant with T1D did not identify as White or White and another race.

As expected, participants with T1D had significantly higher values for fasting glucose (control 88 ±4 vs T1D 175 ±74 mg/dL, *P* < .001) and HbA1c (control 5.0% ±0.4 vs T1D 8.1% ±1.3, *P* < .001) (Table 2). Participants with T1D also had slightly elevated red blood cell count (control 4.4 ±0.2 vs T1D 4.6 ±0.3 M/uL, *P* = .020), decreased sodium (control 139.1 ±1.7 vs T1D 137.3 ±2.3 mmol/L, *P* = .025), and decreased chloride (control 103.1 ±2.1 vs T1D 101.1 ±2.1 mmol/L, *P* = .015) (Table 2). However, these values were still within normal limits. Other metabolites and factors including serum calcium, 25-hydroxyvitamin D, alkaline phosphatase, creatinine, and bicarbonate did not differ significantly between groups (Table 2). Similarly, there were no differences in plasma lipids including triglyceride and cholesterol or in hemoglobin or other blood cell counts (Table 2).

**Table 2.** Hematologic assessments

Parameter	Control (n = 12)	T1D (n = 20)*^a^*	*P*	Reference range
**Fasting glucose (mg/dL)**	**87.8 ± 4.0**	**175.3 ± 74.1** *^b^*	**<.001**	**64–99**
**Hemoglobin A1c (%)**	**5.0 ± 0.4**	**8.1 ± 1.3** *^b^*	**<.001**	**5.1–5.6**
White blood count (K/uL)	6.3 ± 1.7	6.3 ± 1.5	.922	3.6–11.2
**Red blood count (M/uL)**	**4.4 ± 0.2**	**4.6 ± 0.3**	**.020**	**3.63–4.92**
Hemoglobin (g/dL)	13.0 ± 0.5	13.5 ± 0.9	.116	11.9–15.5
Hematocrit (%)	38.7 ± 1.0	40.0 ± 2.3	.072	36.1–44.3
Calcium (mg/dL)	9.6 ± 0.3	9.7 ± 0.3	.337	8.6–11.0
Albumin (g/dL)	4.7 ± 0.2	4.6 ± 0.3	.278	2.5–5.0
Vitamin D (ng/mL)	23.1 ± 4.6*^b^*	27.1 ± 13.4*^b^*	.683	30.0–100.0
Creatinine (mg/dL)	0.63 ± 0.08	0.65 ± 0.10	.601	0.20–0.80
**Sodium (mmol/L)**	**139.1 ± 1.7**	**137.3 ± 2.3**	**.025**	**135–145**
**Chloride (mmol/L)**	**103.1 ± 2.1**	**101.1 ± 2.1**	**.015**	**95–107**
Urea nitrogen (mg/dL)	11.4 ± 3.4	11.8 ± 2.7	.759	9–18
CO_2_ Content (mmol/L)	23. 5 ± 1.7	23.7 ± 1.5	.798	17–32
Triglyceride (mg/dL)	68.1 ± 21.6	86.4 ± 90.4	.626	0–149
Total cholesterol (mg/dL)	163.8 ± 26.6	166.5 ± 38.3	.953	0–169
Direct HDL cholesterol (mg/dL)	62.4 ± 15.8	60.7 ± 13.4	.738	>39
Friedewald LDL cholesterol (mg/dL)	87.7 ± 20.4	90.5 ± 33.2	1.000	0–129
Total protein (g/dL)	7.1 ± 0.3	7.0 ± 0.4	.558	6.1–8.4
Total bilirubin (mg/dL)	0.5 ± 0.2	0.5 ± 0.2	.739	0.30–1.10
Alkaline phosphatase, Total (IU/L)	171.3 ± 109.3	138.0 ± 60.8	.276	70–550
Aspartate transaminase (IU/L)	16.8 ± 2.7	19.7 ± 10.2	.938	10–50
Alanine transaminase (IU/L)	10.1 ± 2.5	12.2 ± 3.6	.082	6–53
Potassium (mmol/L)	4.5 ± 0.4	4.5 ± 0.3	.909	3.3–5.1
Mean corpuscular volume (fL)	87.6 ± 4.2	86.2 ± 3.5	.192	80.0–97.6
Mean corpuscular hemoglobin (pg)	29.6 ± 1.8	29.1 ± 1.5	.391	26.7–33.7
Mean corpuscular hemoglobin concentration (g/dL)	33.7 ± 0.9	33.7 ± 0.6	.950	32.7–35.5
RBC dist width (%)	13.3 ± 0.8	13.4 ± 0.9	.654	12.3–17.0
Platelet count (K/uL)	264.6 ± 42.6	287.8 ± 34.3	.102	140–440
Mean platelet volume (fL)	8.4 ± 1.0	9.0 ± 0.9	.096	6.8–10.4
Neutrophil %	57.6 ± 10.9	54.9 ± 7.6	.416	38.7–74.5
Lymphocyte %	32.0 ± 9.8	34.1 ± 7.3	.480	20.0–54.3
Monocytes %	7.7 ± 2.1	7.5 ± 1.4	.803	4.3–13.5
Eosinophil %	2.1 ± 1.3	2.8 ± 1.6	.179	0.0–6.0
Basophil %	0.7 ± 0.3	0.6 ± 0.2	.694	0.0–3.0
Absolute neutrophil (K/uL)	3.7 ± 1.6	3.5 ± 1.3	.984	1.8–6.6
Absolute lymphocyte (K/uL)	1.9 ± 0.5	2.1 ± 0.4	.416	0.3–3.3
Absolute monocyte (K/uL)	0.5 ± 0.1	0.5 ± 0.1	.703	0.2–1.2
Absolute eosinophil (K/uL)	0.1 ± 0.1	0.2 ± 0.1	.101	0.0–0.5
Absolute basophil (K/uL)	0.0 ± 0.0	0.0 ± 0.1	.276	0.0–0.2
Nucleated RBCs % (/100WBC)	0.1 ± 0.1	0.1 ± 0.1	.52	0.0–0.4

Data are expressed as mean ± SD. Values of *P* were calculated by 2-tailed *t*-test and Mann–Whitney U-test. Significant *P* < .05 values are shown in bold; trending *P* < .01 in italicized bold. Data presented as mean ± SD.

Abbreviations: HDL, high-density lipoprotein; LDL, low-density lipoprotein; RBC, red blood cell.

^*a*^One participant from the total T1D group (n = 21) did not tolerate blood draw. Reference range values are provided for established normal limits within the population.

^*b*^Value outside of reference range.

### Adolescent Girls With T1D Have Decreased Trabecular Bone With Variable Changes in Cortical Porosity and Mineral Density

Bone size, microarchitecture, and volumetric BMD were assessed with second-generation HR-pQCT at the distal radius and tibia (XtremeCT-II, 61 µm resolution). To control for differences in body size (Table 1), bone outcomes were adjusted for BMIz as a composite index of height and weight that is scaled for age. There was no difference in adjusted cross-sectional bone size between groups as measured by cortical area, total area, and cortical perimeter (Table 3). However, there were several differences in microarchitectural parameters between groups. Specifically, participants with T1D had decreased total bone volume/trabecular volume at both the distal radius and the distal tibia that was 14.6% lower [95% confidence interval (CI) −31.9% to 2.7%, *P*-adj = .095] or 12.6% lower (95% CI −23.0% to −2.4%, *P*-adj = .017) than control participants, respectively (Table 3). This difference was due to decreased trabecular thickness at both sites (−8.3% radius, 95% CI −14.3% to −2.6%, *P*-adj = .007; −7.5% tibia, 95% CI −14.3% to −0.8%, *P*-adj = .034) with no difference in trabecular number (Table 3). Changes in cortical microarchitecture were also noted. Specifically, we observed a significant 52.9% decrease in cortical porosity (95% CI −93.6% to −12.2%, *P*-adj = .012) and an 8.6% increase in cortical volumetric bone mineral density (vBMD) (95% CI 1.2% to 15%, *P*-adj = .024) at the tibia of subjects with T1D (Table 3). There were no microarchitectural differences in the cortical bone at the radius. Finite element analysis-based estimates of bone strength were not significantly different between groups.

**Table 3.** HRpQCT of the ultradistal radius and tibia

Site	Parameter	Control (n = 12)	T1D (n = 21)	*P-adj*	Estimate T1D vs control (adj %)	β-coefficient	95% CI
Radius	***BV/TV (%)***	***21.8 ± 5.8***	***20.6 ± 4.8***	***.095***	***−14.6***	***−3.4***	***(−7.42, 0.63)***
	Tb. N (1/mm)	1.360 ± 0.143	1.385 ± 0.197	.958	−0.3	−0.0039	(−0.154, 0.147)
	**Tb. Th (mm)**	**0.225 ± 0.022**	**0.215 ± 0.016**	**.007**	**−8.3**	**−0.0192**	**(−0.033, −0.006)**
	***Tb. vBMD (mgHA/ccm)***	***154.1 ± 39.4***	***143.8 ± 31.8***	***.077***	***−14.9***	***−24.3***	***(−51.5, 2.8)***
	Ct. Th (mm)	1.106 ± 0.309	1.212 ± 0.231	.407	8.2	0.091	(−0.130, 0.312)
	Ct. Po (%)	0.5 ± 0.4	0.7 ± 0.7	.915	−4.0	−0.03	(−0.51, 0.46)
	Ct. vBMD (mgHA/ccm)	799.5 ± 98.5	835.6 ± 87.0	.197	6.3	49.5	(−27.1, 126.1)
	Tt. Ar (mm^2^)	190.0 ± 14.6	194.2 ± 32.7	.862	1.0	2.0	(−21.4, 25.4)
	Tb. Ar (mm^2^)	141.0 ± 18.7	140.3 ± 28.9	.857	−1.4	−1.9	(−23.7, 19.9)
	Ct. Pm (mm)	55.2 ± 2.6	56.1 ± 4.9	.737	1.1	0.6	(−3.0, 4.2)
	Ct. Ar (mm^2^)	51.8 ± 13.6	58.3 ± 12.9	.317	10.6	5.5	(−5.6, 16.7)
	Failure load (n)	3281.2 ± 934	3586.6 ± 767	.640	4.8	160.4	(−533.6, 854.3)
	Stiffness (N/mm)	58 667 ± 16 913	63 638 ± 13 713	.712	3.8	2277	(−10192, 14747)
	Apparent modulus of elasticity (N/mm^2^)	2449.0 ± 766	2634.0 ± 458	.602	5.1	127.9	(−367.3, 623.1)
Tibia	**BV/TV (%)**	**27.0 ± 4.6**	**25.1 ± 3.2**	**.017**	**−12.8**	**−3.6**	**(−6.47, −0.68)**
	Tb. N (1/mm)	1.291 ± 0.156	1.345 ± 0.218	.815	1.5	0.0191	(−0.147, 0.185)
	**Tb. Th (mm)**	**0.261 ± 0.025**	**0.248 ± 0.021**	**.034**	**−7.5**	**−0.0199**	**(−0.038, −0.002)**
	**Tb. vBMD (mgHA/ccm)**	**178.8 ± 32.1**	**166.5 ± 24.3**	**.022**	**−13.3**	**−24.8**	**(−45.7, −3.9)**
	Ct. Th (mm)	1.401 ± 0.382	1.403 ± 0.245	.319	−7.9	−0.117	(−0.353, 0.119)
	**Ct. Po (%)**	**2.1 ± 1.6**	**1.3 ± 1.1**	**.012**	**−52.9**	**−1.3**	**(−2.3, −0.3)**
	Tt. Ar (mm^2^)	592.2 ± 58.9	601.6 ± 112.1	.790	−1.7	−10.6	(−90.9, 69.7)
	Tb. Ar (mm^2^)	484.4 ± 51.5	490.3 ± 107.9	.911	−0.9	−4.3	(−81.8, 73.2)
	Ct. Pm (mm)	93.9 ± 4.7	94.6 ± 8.6	.756	−1.0	−1.0	(−7.1, 5.2)
	Ct. Ar (mm^2^)	112.6 ± 29.3	116.2 ± 19.2	.478	−5.3	−6.4	(−24.4, 19.9)
	**Ct. vBMD (mgHA/ccm)**	**819.9 ± 86.5**	**867.1 ± 65.4**	**.024**	**8.6**	**69.6**	**(9.9, 129.4)**
	Failure load (n)	9577 ± 2579	9498 ± 1717	.242	−9.1	−921.1	(−2496.2, 653.9)
	Stiffness (N/mm)	177 273 ± 48 743	173 772 ± 32 007	.190	−10.4	−19 400	(−48935, 10135)
	Apparent modulus of elasticity (N/mm^2^)	2625 ± 611	2605 ± 447	.400	−6.3	−171	(−579, 238)

Absolute data are expressed as mean ± SD. Values of *P*, estimated percent change in T1D vs control, and β-coefficient were calculated by linear regression adjusted for body mass index Z score to consider age-adjusted variability in body size. Significant *P* < .05 values are shown in bold, trending *P* < .01 in italicized bold. β-coefficients in linear regression models describe the change in outcome based on the change in predictor. In our data, for example, the diagnosis of T1D for ≥ 5 years (predictor) was associated with a significant likelihood of a −0.0192 mm change in radius trabecular thickness (outcome) relative to the adjusted mean of 0.231 mm in the control group (*P*-adj = .007). This means that trabecular thickness in adolescent girls with T1D is predicted to be 8.3% lower than what would be expected in a nondiabetic person of comparable body size (−0.0192/0.231 = −0.083 = −8.3%).

Abbreviations: Ar, area; BV/TV, total bone volume/trabecular volume; CI, confidence interval; Ct, cortical; Pm, perimeter; Po, porosity; Tb, trabecular; Th, thickness; T1D, type 1 diabetes; vBMD, volumetric bone mineral density.

### Adolescent Girls With T1D Have Evidence of Decreased Bone Formation and Turnover

**Figure 1.**
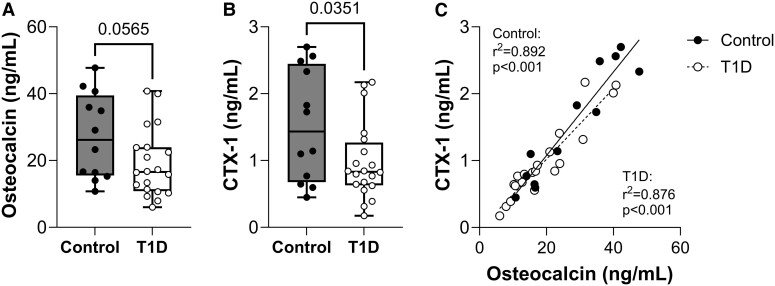
Circulating biomarkers of bone turnover are decreased in adolescent girls with T1D. (A) Serum osteocalcin, a measure of osteoblast function. (B) Serum CTX-1, a marker of osteoclast activity. (C) Correlation between osteocalcin and CTX-1 in control participants and those with T1D. Abbreviations: CTX-1, collagen cross-linked C-telopeptide-1; TD1, type 1 diabetes.

Parameters of bone forming and bone resorbing cells were evaluated using serum biomarkers. The circulating concentration of osteocalcin, a biomarker of osteoblast number and function, was 30% lower in participants with T1D (control 27.2 ± 12.6 vs T1D 19.1 ± 10.4 ng/mL, *P* = .057) (Fig.1A). Similarly, CTX-1, a marker of osteoclast activity, was 36% lower in T1D (control 1.527 ± 0.845 vs T1D 0.975 ± 0.572 ng/mL, *P* = .035) (Fig.1B). Circulating concentrations of bone formation and resorption biomarkers were highly correlated in both control (r^2^ = 0.892, *P* < .001) and T1D groups (r^2^ = 0.876, *P* < .001) (Fig.1C).

### DXA Fails to Detect Early Changes in Bone in Participants With T1D

**Figure 2.**
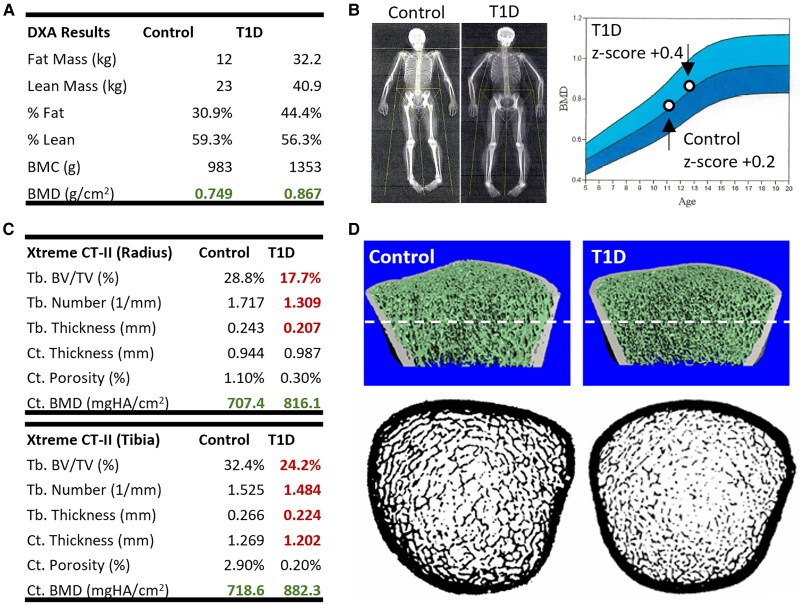
Assessment of bone by DXA vs HRpQCT. Representative example of the comparison in outcomes between (A, B) DXA and (C, D) HRpQCT with Xtreme CT-II. The same 2 participants are used for both studies, 1 from the control group and 1 with T1D. (A, B) By DXA, both participants were found to score within the normal range for BMD. (C) However, by HRpQCT, substantial underlying decreases in trabecular bone microarchitecture were detected. (D) 3D reconstruction of the bone scan (top) and corresponding 2D slice (dotted line, bottom) show deterioration of trabecular bone in the tibia. Abbreviations: BV/TV, bone volume/total volume; BMD, bone mineral density; DXA, dual-energy x-ray absorptiometry; HRpQCT, high-resolution peripheral quantitative computed tomograpy; T1D, type 1 diabetes.

Areal BMD was also measured by DXA. There were no significant differences in absolute or BMIz-adjusted arial BMD by DXA at all sites examined (Table 4). This included subtotal BMD (whole body less head), subtotal BMD Z-score, thoracic spine BMD, lumbar spine BMD, pelvis BMD, nondominant arm BMD, and nondominant leg BMD as quantified regionally on the whole-body scan. This finding emphasizes the inadequate sensitivity of classical DXA to detect early changes in T1D bone that may already be prominent by HR-pQCT. A visual example is provided in Fig. 2. In this case, both the control and T1D participant had a normal Z-score by DXA (Fig.2A and 2B). However, HR-pQCT scans of the same participants revealed an underlying 25% and 39% decrease in trabecular bone volume fraction in the tibia and radius, respectively, of the T1D participant (Fig.2C and 2D).

**Table 4.** Bone mineral density as assessed by DXA

DXA parameter	Control (n = 12)	T1D (n = 21)	*P*	*P-adj*
Thoracic spine BMD (g/cm^2^)	0.801 ± 0.122	0.841 ± 0.115	.343	.703
Lumbar spine BMD (g/cm^2^)	0.963 ± 0.125	1.034 ± 0.116	.108	.178
Pelvis BMD (g/cm^2^)	1.145 ± 0.149	1.205 ± 0.139	.251	0.933
Nondominant arm BMD (g/cm^2^)	0.664 ± 0.079	0.692 ± 0.078	.338	.687
Nondominant leg BMD (g/cm^2^)	0.997 ± 0.123	1.029 ± 0.089	.382	.904
Subtotal BMD (g/cm^2^)	0.946 ± 0.098	1.008 ± 0.084	.062	.129
Subtotal BMD z-score	−0.5 ± 1.4	−0.2 ± 0.8	.547	.591

Absolute data are expressed as mean ± SD. *P* values from comparison of absolute data are given as p. *P*-values calculated by linear regression after adjustment for body mass index Z score to consider age-adjusted variability in body size are given as *P*-adj.

Abbreviations: BMD, bone mineral density; DXA, dual-energy X-ray absorptiometry.

### Changes in Bone With T1D Present Prior to the Onset of Clinical Neuropathy

A primary goal of our study was to determine if the onset of bone loss and osteoblast suppression in adolescents with T1D occurs before or after clinical signs of neuropathy. To do this, we used the MNSI and a standard monofilament exam. The MNSI can reliably detect DPN in children and adults with T1D, as evidenced by use in large multicenter trials including DCCT/EDIC (26), the SEARCH for Diabetes in Youth Study (14), and the TODAY study for youth with T2D (13). Reduced or absent response to the monofilament exam is also suggestive of DPN but has more limited sensitivity (27). All participants in our cohort had normal monofilament exams (Table 5). Two participants in our cohort with T1D scored at the MNSI cutoff (exam score = 2), for a prevalence of 9.5% (Table 5). The values for the bone microarchitectural parameters and bone turnover biomarkers of the 2 individuals with DPN were distributed throughout the range of the T1D participants overall (Table 6). Either controlling for or dropping these values from our models failed to explain the more pervasive changes in bone or bone cell function in participants with T1D.

**Table 5.** Diagnosis of diabetic peripheral neuropathy by the MNSI and monofilament exam

Assessment	T1D (n = 21)	Reference range	N positive (%)
MNSI survey score	0.67 ± 0.91	>4	0
MNSI clinical exam score	0.43 ± 0.64	≥2	2 (9.5)
Monofilament exam	All normal	≥8	0

Youth with type 1 diabetes are considered positive for diabetic peripheral neuropathy based on a survey score > 4 (of 15 total points) and/or an exam score ≥ 2 (of 8 total points) and/or an abnormal monofilament exam. Data are expressed as mean ± SD.

Abbreviations: MNSI, Michigan Neuropathy Screening Instrument; T1D, type 1 diabetes.

**Table 6.** HR-pQCT and bone biomarker values for T1D + DPN.

Parameter	All T1D (n = 21, min to max)	T1D + DPN (n = 2)
Osteocalcin (ng/mL)	6.0-40.8	16.4, 9.3
CTX (ng/mL)	0.173-2.171	0.556, 0.388
Radius BV/TV (%)	10.7-28.2	10.7, 24.0
Radius Tb.N (1/mm)	0.923-1.764	0.923, 1.352
Radius Tb.Th (mm)	0.183-0.242	0.183, 0.242
Radius Ct.Th (mm)	0.801-1.502	1.495, 1.432
Radius Ct.Po. (%)	0-2.7	0, 1.5
Radius failure load (n)	2172-5625	3707, 2956
Tibia BV/TV (%)	18.9-30.9	21.2, 28.3
Tibia Tb.N (1/mm)	0.978-1.739	1.179, 1.005
Tibia Tb.Th (mm)	0.210-0.312	0.254, 0.312
Tibia Ct.Th (mm)	1.032-2.016	1.416, 2.016
Tibia Ct.Po. (%)	0.2-3.2	0.4, 3.2
Tibia failure load (n)	6757-12 340	8418, 12124

Absolute data expressed from minimum to maximum recorded values (all T1D participants) vs absolute values for the 2 participants with DPN.

Abbreviations: Ar, area; BV/TV, total bone volume/trabecular volume; Ct, cortical; DPN, diabetic peripheral neuropathy; Po, porosity; Pm, perimeter; Tb, trabecular; Th, thickness; T1D, type 1 diabetes; vBMD, volumetric bone mineral density.

## Discussion

We found that adolescent girls with T1D had decreased trabecular bone mass, decreased tibial cortical porosity, and decreased markers of bone formation and turnover, independent of neuropathy status. This aligns with recent research in mice showing that suppression of osteoblast function occurs rapidly after the onset of T1D and that this is completely independent of DPN (28). Overall, our findings suggest that early identification and management of diabetic bone disease, independent of neuropathy status, is warranted to prevent fracture and related comorbidities later in life. In adults with T1D, the relative risk of hip fracture is 4.9-fold higher than the general population (95% CI 3.06–7.95) and, unlike most osteoporotic fractures, risk is increased in those under 65 years of age (29). Odds of fracture are highest in individuals with poor glycemic control, severe hypoglycemia, celiac disease, or a history of smoking (30). Statistical power limited our ability to corroborate glycemic control with bone outcomes. However, the differences in fracture rate in our study (48% in T1D vs 33% in controls, hazard ratio of 1.45) mirror what has been found in larger cohorts with a hazard ratio for all fractures of 1.35 in females with T1D at ≤ 19 years of age (95% CI 1.12–1.63) (4).

Altered bone microarchitecture in persons with T1D may at least partially explain the increased fracture risk. Four studies including the current publication have examined skeletal microarchitecture in youth with T1D by HR-pQCT (2, 24, 31). Altogether, this has included 189 participants with T1D and 153 controls from 6 to 18 years of age. The 3 prior studies from 2020 used the first-generation HR-pQCT (82 um resolution) while this study used the HR-pQCT-II (61 um resolution). Consistent findings include deficits in trabecular bone volume and microarchitecture in both male and female youth with T1D, most commonly due to decreased trabecular thickness. The mean trabecular thickness in our cohort was 236 µm, which is higher than what has been reported with first-generation HR-pQCT (∼90 µm). Our results, however, align with prior measures of trabecular thickness in human bone as measured by micro computed tomography and histology (32-34), and the difference is likely due to underlying improvements in resolution in the second-generation HR-pQCT. Similarly, our data with higher resolution HR-pQCT identified lower cortical porosity and higher cortical vBMD with T1D in weight-bearing bone. Though cortical porosity is generally detrimental in adults, physiologic cortical porosity is common in rapidly growing children and in athletes, driven by increased bone anabolic activity. Lower porosity in our study may reflect decreased anabolic activity or low bone turnover in adolescents with T1D. Though not significant in our study, likely due to insufficient power for this metric, decreased predicted bone strength is also a common finding by HR-pQCT in adolescents with T1D (2, 24, 31). Emerging profiles for compartment- and site-specific changes in cortical and trabecular vBMD in adolescents with T1D may have contributed to the predicted biomechanical properties of our population, and this point warrants additional future investigation.

Radiographic findings of bone often correlate with bone cell dysfunction. Consistent with this, participants with T1D presented with an average ∼30% suppression of markers of both osteoblast and osteoclast function, indicating relative deficiencies in bone turnover. When considered individually, some even had values 80% to 90% below the control mean (Fig. 1). Our findings of low bone formation and turnover among persons with T1D are consistent with prior reports (35-37). The skeleton is estimated to fully renew itself once every 10-years (38). If this is delayed by impaired new bone formation, decreased resorption, or a combination of both, substantial additional damage can accumulate over time that predisposes to fracture. For example, a 30% decrease in bone turnover on a yearly basis could extend the time to skeletal renewal from 10 to 14.3 years, while a 90% decrease could increase this to 100 years. In addition to T1D-associated deficiencies in bone formation, any delays in turnover would likely contribute to the accumulation of structural deficits and microcracks in addition to matrix modifications such as advanced glycation end products that can stiffen and weaken individual collagen fibers (39).

The potential for functional relationships between bone health and DPN in T1D at different stages of disease remains an active area of investigation (12, 28). Like fracture, increased risk for neuropathy in persons with T1D begins in childhood. In a study of children between 2 and 16 years of age with T1D, 18% had subclinical neuropathy by measures of nerve conduction (40). Larger cohorts based on the MNSI reveal that clinical signs of DPN are present in 7% of adolescents with T1D and 22% to 39% of adolescents with T2D (13, 14). The prevalence of DPN based on the MNSI in our study (9.5%) is well-aligned with prior work. However, this limited onset of DPN failed to explain the significant decreases in bone microarchitecture and bone cell biomarkers that were prevalent in participants with T1D. Emerging evidence in rodents also reveals that subclinical neuropathy is unlikely to play a role in the onset and early progression of diabetic bone disease (28), suggesting that these systems function relatively independently in the earlier phases of T1D. Overall, the clinical correlations between neuropathy and fracture risk seem to become more important later in the course of the disease when neuropathologies lead to muscle weakness, altered gait, and increased risk of falls—all of which can predispose to fracture (12).

Altogether, this work supports the identification of strategies to increase osteoblast function and bone turnover to support lifelong skeletal renewal and bone strength in those living with T1D. In 2024, the ADA published an updated subsection on bone health for adults with diabetes and now recommends that fracture risk assessment be a part of routine clinical care (41, 42). Similarly, the 2022 Clinical Practice Consensus Guidelines from the International Society for Pediatric and Adolescent Diabetes suggest that lifelong impairments to bone acquisition and turnover in T1D begin in adolescence and childhood, while calling for more research to define suitable screening tools and interventions (43). For example, though whole-body DXA seems to be consistently inadequate to detect early disease, possibly masked by early increases in cortical BMD in those with T1D, it is interesting to speculate whether a more targeted, trabecular bone score could be a valuable adjunct for clinical assessment in pediatric populations (44). At present, both pediatric and adult diabetic bone disease are considered secondary causes of osteoporosis and assessed with bone density. Yet, just as the careful distinction between vitamin D-deficient rickets and renal osteodystrophy has substantial clinical implications, attention to how diabetic bone disease differs from postmenopausal osteoporosis could foster significant changes to comprehensive diabetes care. A high correlation between biomarkers of osteoblast and osteoclast function suggests persistent coupling of skeletal cells in adolescents with T1D. This is very different from postmenopausal osteoporosis, where excess osteoclast activity is uncoupled from osteoblasts and can be treated with antiresorptive medications. Exercise, as recommended by the ADA, represents a logical first-line treatment to promote bone cell function. However, this is unlikely to be enough to truly restore healthy levels of bone turnover throughout the body. Additional therapies that reactivate skeletal cells are likely to have substantial benefit.

### Other Findings and Considerations

The secondary finding that plasma sodium was lower in our participants with T1D may have implications yet unexplored. It is well known that glucose acts as an osmole that draws water from the intracellular fluid into the extracellular fluid. It is not unexpected, therefore, that the patients in our cohort with frequent hyperglycemia secondary to T1D had significantly lower serum sodium and chloride levels compared to controls. However, the association of hyponatremia with gait instability, osteoporosis, increased falls, and bone fractures (45-47), as well as recent speculation that hyponatremia could be contributing to bone disease in patients through mechanisms of altered osteoclast and osteoblast activity independent of osmolality (48), prompts us to highlight the result. Slowed nerve conduction and increased risk for the development of peripheral neuropathy in patients with concurrent diabetes and low serum sodium levels (even in the normal range) have also been reported in the literature (49, 50). Future work is needed to explore the possibility that dilutional hyponatremia from hyperglycemia could contribute to increased fracture risk in diabetes mellitus.

### Strengths and Limitations

This is the first study to systematically and simultaneously quantify neuropathy and bone outcomes in youth with T1D. Limitations include the small size of the study. Future work will focus on expanding the size of our cohorts, including males, and prospectively evaluating changes in bone metabolism and nerve function. Participants with T1D in this study also had better average glycemic control relative to nationwide cohorts (8.1% average HbA1c vs 9.3%), which may have limited the severity of diabetes-associated pathologies (51). Last, assessment of neuropathy with the MNSI has high sensitivity and specificity for the identification of clinical DPN and has been used in large pediatric cohorts with both T1D and T2D (13, 14) but is unable to detect subclinical neuropathy. Alternate techniques such as motor and sensory nerve conduction studies, heart rate variability, and skin biopsy may enhance the sensitivity of future assessments.

## Conclusions

Using second-generation HR-pQCT imaging with enhanced precision, we found that adolescent girls with 8.5 ±3.2 years of T1D had clear evidence of compromised trabecular microarchitecture across skeletal sites. Our data also demonstrate early changes in cortical microarchitecture in load-bearing bone and show evidence of decreased bone formation and turnover based on circulating biomarkers. The relatively low prevalence of clinical DPN in our cohort despite significant alterations in bone parameters suggests that the onset of neuropathy is not necessary for bone deterioration during the early stages of T1D.

## Data Availability

Original data generated and analyzed during this study are included in this published article. The full deidentified underlying dataset is available for request at DOI 10.17632/yszdzvn94n.1 as part of the Washington University Institutional Repository. This was completed with the permission and oversight of the Washington University Institutional Review Board.
